# Supplementary material for: Characterization and Transcriptome Analysis Reveal Abnormal Pollen Germination in Cytoplasmic Male Sterile Tomato
Source: Int J Mol Sci. 2025 Aug 28;26(17):8337. doi: 10.3390/ijms26178337 (PMC12428091; doi:10.3390/ijms26178337)
Supplement: Supplementary file 1 [file ijms-26-08337-s001.zip › supplementaryFigs.pptx]

## Slide 1
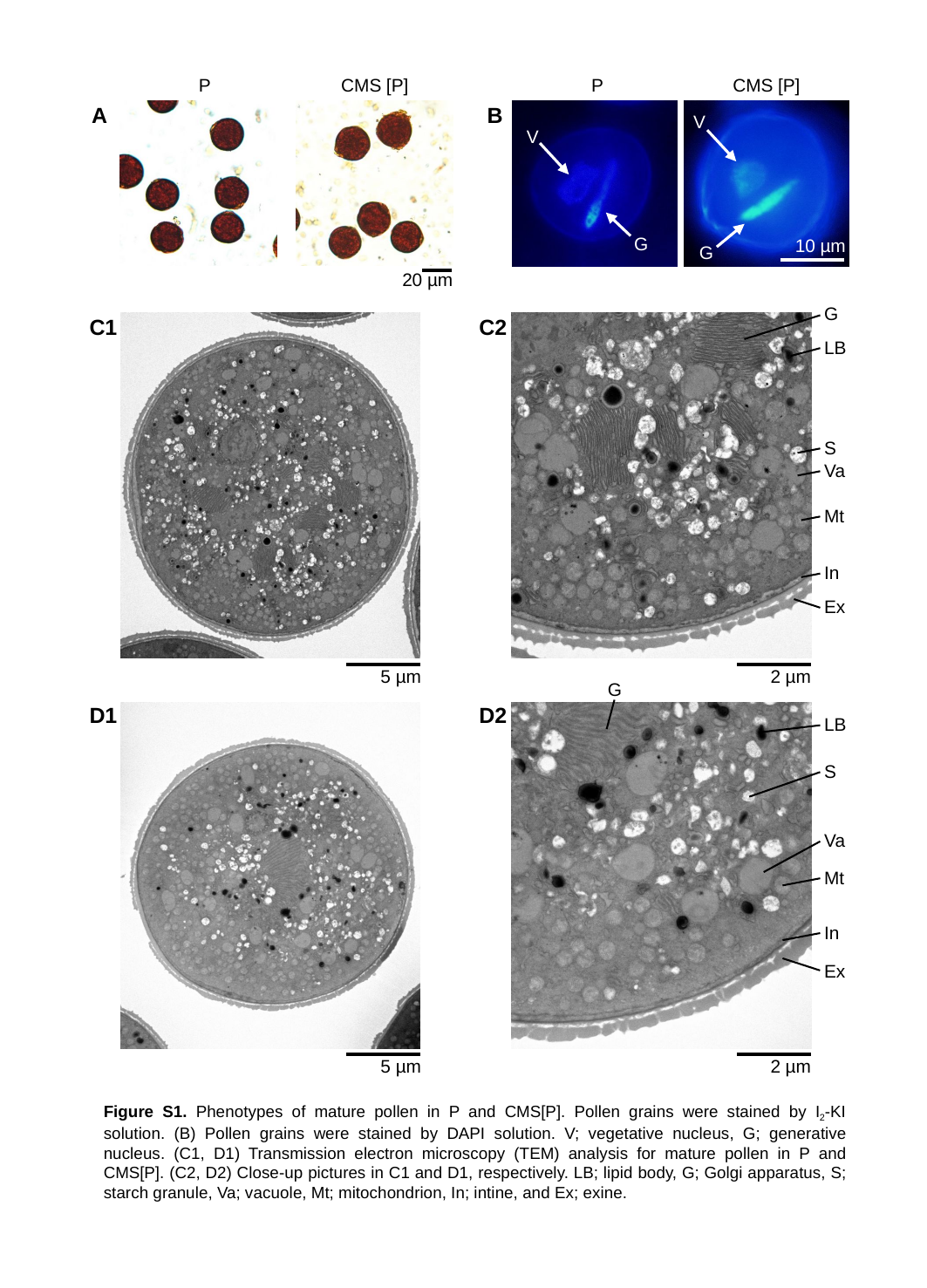

P
CMS [P]
P
CMS [P]
A
B
V
V
G
10 µm
G
20 µm
G
C1
C2
LB
S
Va
Mt
In
Ex
5 µm
2 µm
G
D1
D2
LB
S
Va
Mt
In
Ex
5 µm
2 µm
Figure S1. Phenotypes of mature pollen in P and CMS[P]. Pollen grains were stained by I2-KI solution. (B) Pollen grains were stained by DAPI solution. V; vegetative nucleus, G; generative nucleus. (C1, D1) Transmission electron microscopy (TEM) analysis for mature pollen in P and CMS[P]. (C2, D2) Close-up pictures in C1 and D1, respectively. LB; lipid body, G; Golgi apparatus, S; starch granule, Va; vacuole, Mt; mitochondrion, In; intine, and Ex; exine.

## Slide 2
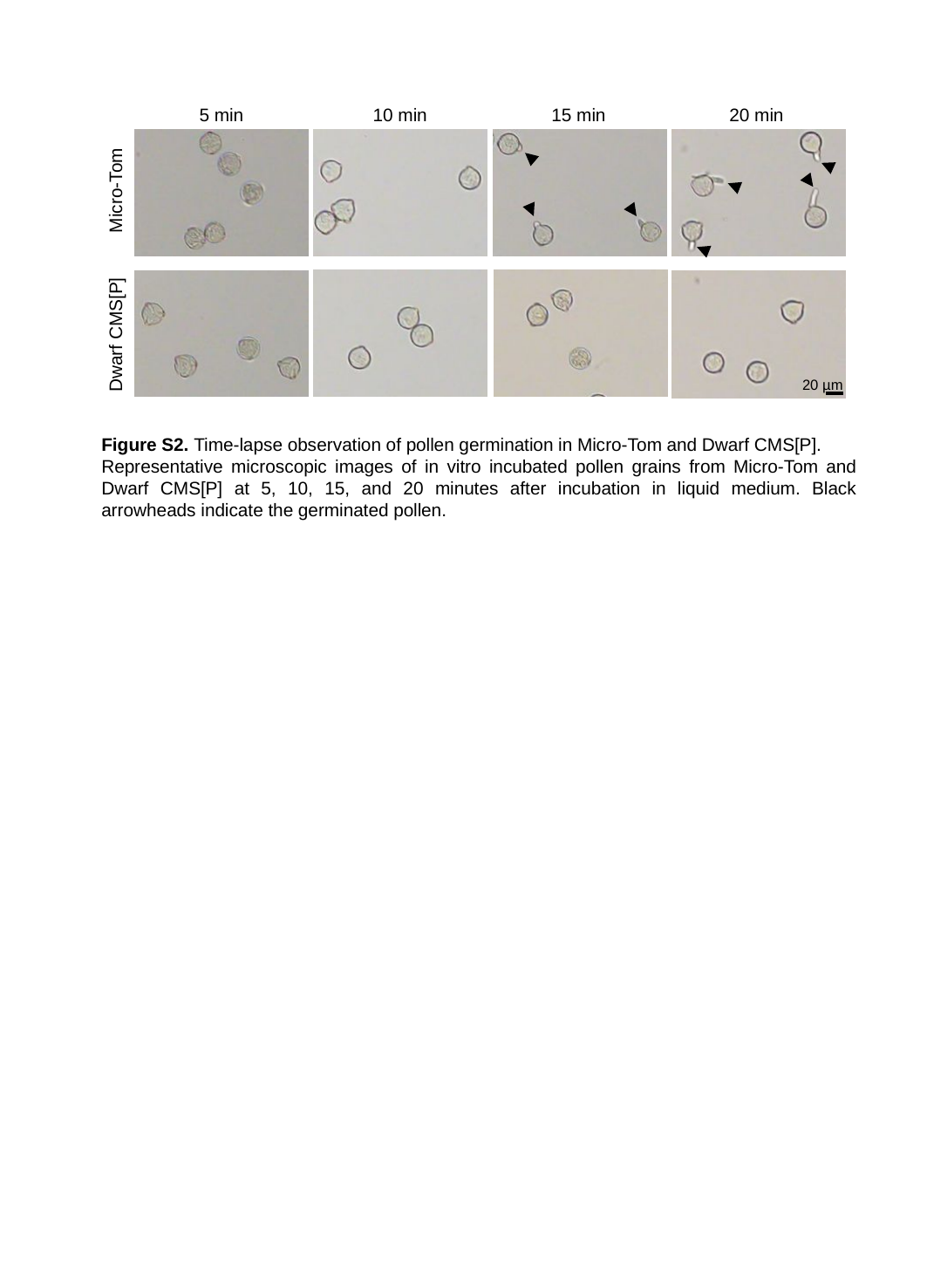

5 min
10 min
15 min
20 min
Micro-Tom
Dwarf CMS[P]
20 µm
Figure S2. Time-lapse observation of pollen germination in Micro-Tom and Dwarf CMS[P].
Representative microscopic images of in vitro incubated pollen grains from Micro-Tom and Dwarf CMS[P] at 5, 10, 15, and 20 minutes after incubation in liquid medium. Black arrowheads indicate the germinated pollen.

## Slide 3
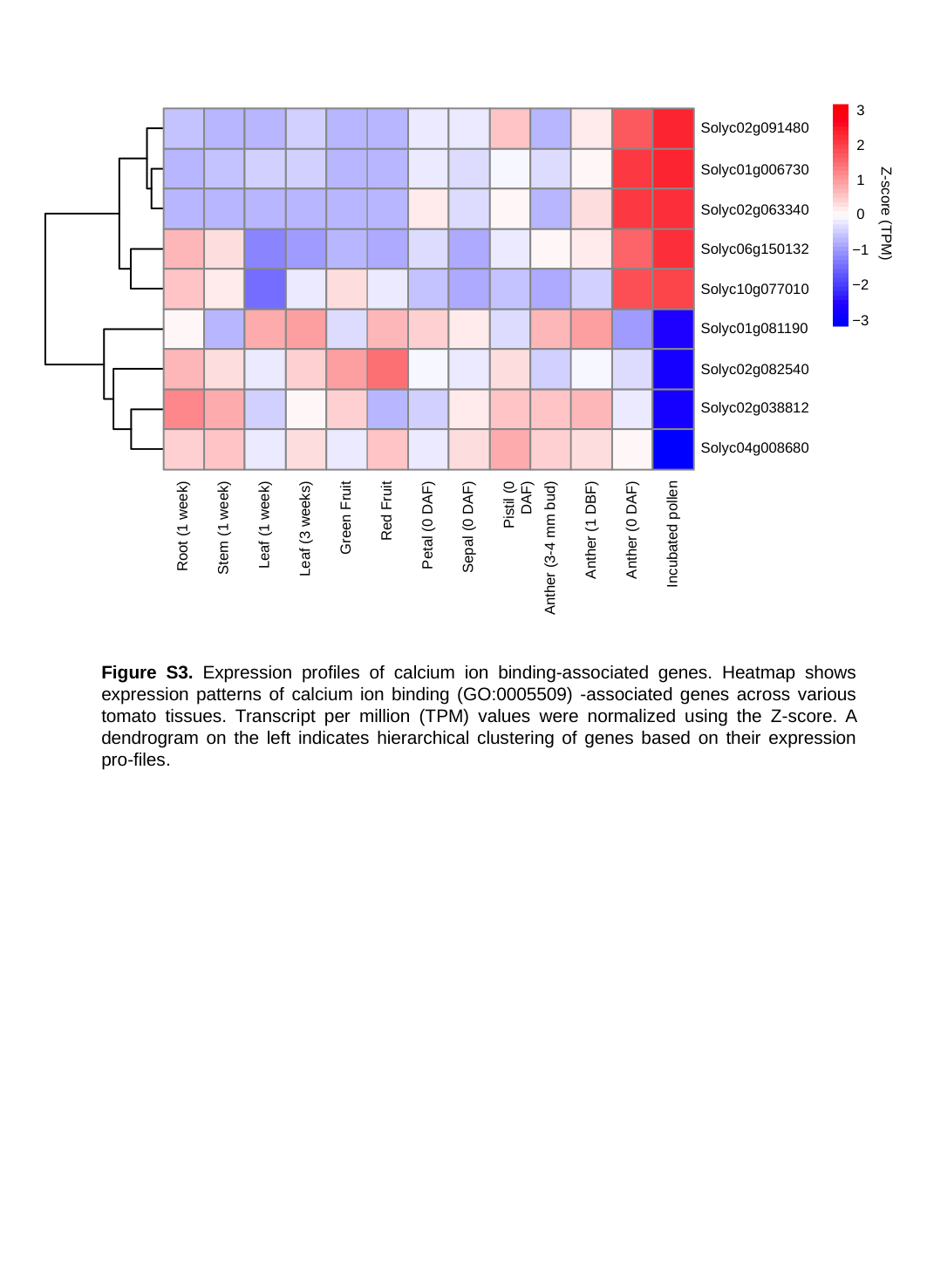

3
Solyc02g091480
2
Solyc01g006730
1
Solyc02g063340
0
Z-score (TPM)
Solyc06g150132
−1
−2
Solyc10g077010
−3
Solyc01g081190
Solyc02g082540
Solyc02g038812
Solyc04g008680
Green Fruit
Red Fruit
Pistil (0 DAF)
Root (1 week)
Leaf (1 week)
Leaf (3 weeks)
Sepal (0 DAF)
Petal (0 DAF)
Stem (1 week)
Anther (3-4 mm bud)
Anther (1 DBF)
Anther (0 DAF)
Incubated pollen
Figure S3. Expression profiles of calcium ion binding-associated genes. Heatmap shows expression patterns of calcium ion binding (GO:0005509) -associated genes across various tomato tissues. Transcript per million (TPM) values were normalized using the Z-score. A dendrogram on the left indicates hierarchical clustering of genes based on their expression pro-files.
